# Culturing of female bladder bacteria reveals an interconnected urogenital microbiota

**DOI:** 10.1038/s41467-018-03968-5

**Published:** 2018-04-19

**Authors:** Krystal Thomas-White, Samuel C. Forster, Nitin Kumar, Michelle Van Kuiken, Catherine Putonti, Mark D. Stares, Evann E. Hilt, Travis K. Price, Alan J. Wolfe, Trevor D. Lawley

**Affiliations:** 1 Department of Microbiology and Immunology, Stritch School of Medicine, Loyola University Chicago, Maywood, IL 60153 USA; 2 Host-Microbiota Interactions Laboratory, Wellcome Sanger Institute, Hinxton, CB10 1SA UK; 3 Centre for Innate Immunity and Infectious Diseases, Hudson Institute of Medical Research, Clayton, VIC 3168 Australia; 4 Department of Molecular and Translational Sciences, Monash University, Clayton, VIC 3800 Australia; 5 Department of Urology, Stritch School of Medicine, Loyola University Chicago, Maywood, IL 60153 USA; 6 Department of Biology, Loyola University Chicago, Chicago, IL 60660 USA; 7 Department of Computer Science, Loyola University Chicago, Chicago, IL 60660 USA; 8 Bioinformatics Program, Loyola University Chicago, Chicago, IL 60660 USA

## Abstract

Metagenomic analyses have indicated that the female bladder harbors an indigenous microbiota. However, there are few cultured reference strains with sequenced genomes available for functional and experimental analyses. Here we isolate and genome-sequence 149 bacterial strains from catheterized urine of 77 women. This culture collection spans 78 species, representing approximately two thirds of the bacterial diversity within the sampled bladders, including Proteobacteria, Actinobacteria, and Firmicutes. Detailed genomic and functional comparison of the bladder microbiota to the gastrointestinal and vaginal microbiotas demonstrates similar vaginal and bladder microbiota, with functional capacities that are distinct from those observed in the gastrointestinal microbiota. Whole-genome phylogenetic analysis of bacterial strains isolated from the vagina and bladder in the same women identifies highly similar *Escherichia coli*,* Streptococcus anginosus*,* Lactobacillus iners*, and *Lactobacillus crispatus*, suggesting an interlinked female urogenital microbiota that is not only limited to pathogens but is also characteristic of health-associated commensals.

## Abstract (web summary)

The female bladder seems to harbor a poorly characterized indigenous microbiota. Here, the authors isolate and genome-sequence 149 bacterial strains from catheterized urine of 77 women, generating a culture collection representing two thirds of the bacterial diversity within the samples.

## Introduction

Contrary to medical dogma, urine is not sterile, even in asymptomatic individuals^1–5^. For over 60 years, the standard urine culture protocol has represented the primary tool for detecting bacteria in clinical microbiology laboratories. This aerobic protocol was designed to detect causative agents of pyelonephritis^6^ and is particularly effective at detecting abundant *Escherichia coli* (>10^5^ colony-forming unit (CFU)/ml) but little else^7^. Culture-independent analysis of urine obtained by suprapubic aspirate, which bypasses the vulva, vagina, and urethra, demonstrates the presence of bacteria in the bladders of women^1^. The microbial profiles observed in aspirated urine are similar to those in urine obtained by transurethral catheter (TUC), indicating the avoidance of urethral contamination in catheterized samples^1^. We note the difference between TUC, which is transient and not associated with catheter-associated urinary tract infection (CAUTI), and indwelling catheters that are known to harbor biofilms and result in CAUTI. Analyzing urine sampled by TUC, we previously found that the majority of asymptomatic women contain bacterial genera typically not detected by standard urine culture^1,3^. These genera, including *Lactobacillus*, *Gardnerella*, *Streptococcus*, *Staphylococcus*, and *Corynebacterium*, tend to be in low abundance (between 10 and 10^4^ CFU/ml)^3^ and require growth conditions and carbon sources not available in standard urine culture^2,8^. Therefore, we developed an expanded quantitative urine culture (EQUC) protocol that captures a broad range of bacterial taxa^3^.

Previous studies, using EQUC in combination with 16S rRNA sequencing^2,3,9^, suggest the bladder microbiota of asymptomatic women typically contains low bacterial diversity, with increases in diversity indicative of urgency urinary incontinence symptoms and a decreased response to anticholinergic medication^2,9,10^. They also highlight bacterial species (e.g., *Streptococcus anginosus* and *Gardnerella vaginalis*) that are associated with urgency urinary incontinence symptoms and others (e.g., *Lactobacillus crispatus*) that are associated with the lack of lower urinary tract symptoms^2^. Evidence also exists that women with communities dominated by specific *Lactobacillus* species are less likely to develop post-instrumentation and postoperative urinary tract infections (UTIs)^8,11^. However, owing to the lack of bladder-specific bacterial reference genomes, high-resolution taxonomic characterization to the bacterial strain level, functional analysis, and comparison to other microbiota communities remain to be performed.

In the present study, we combined EQUC with large-scale whole-genome sequencing to comprehensively characterize the microbiota composition of the female bladder in symptomatic and asymptomatic peri-menopausal women. We compared this new culture collection to previously collected gut and vaginal collections and identified similarities between the bladder and vagina but not the gut. Finally, we identified highly similar species that reside in both the bladder and vagina of individual women indicative of interconnected urogenital microbiota.

## Results

### The bladder microbiota culture collection

**Fig. 1:**
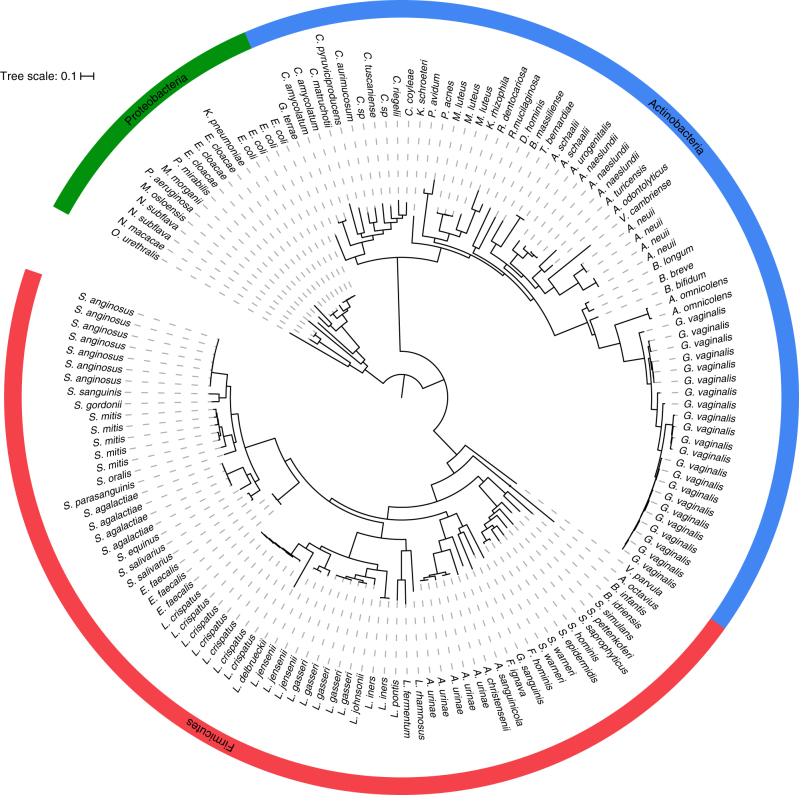
Phylogenetic tree representing diversity of bacteria cultured from the female bladder. A representation of the full bacterial diversity (*n* = 149 isolates) that can be isolated using the expanded quantitative urine culture (EQUC) method from catheterized urine samples (*n* = 77 patients)

Overall, we archived and genome-sequenced 149 isolates representing 3 phyla, 7 classes, 11 orders, 23 families, 36 genera, and 78 species (Fig. 1; Supplementary Data 1). These organisms were isolated from 38 asymptomatic individuals (67 isolates) and 39 symptomatic individuals (82 isolates) (Supplementary Data 1). Uropathogenic species, such as *E. coli*, *Klebsiella pneumoniae*, *Proteus mirabilis, Enterobacter cloaceae*, *Morganella morganii*, and *Pseudomonas aeruginosa*, represented only 7.7% (6/78) of the phylogenetic diversity cultivatable from the bladder. In fact, other than these uropathogens, very few Proteobacteria or even Gram-negative organisms were found. Instead, the largest number of isolated species was from the Gram-positive phyla Firmicutes (47.4%, 37/78) and Actinobacteria (38.5%, 30/78), particularly the families Streptococcaceae (11.0%, 9/78), Lactobacillaceae (11.0%, 9/78), Corynebacteriaceae (10.3%, 8/78), and Actinomycetaceae (10.3%, 8/78). To understand the extent of the total bacterial community represented by this culture collection, we next undertook whole-genome metagenomic sequencing on 12 samples. This analysis suggests that EQUC captures approximately 66.4% of bacterial abundance within the bladder microbiota representing approximately 72.0% of the genera (Supplementary Table 1). The only genera detected by metagenomics without representative strains within our culture collection were anaerobes from the phyla Actinobacteria (*Propionimicrobium*, *Varibaculum*, and *Atopobium*), Firmicutes (*Peptoniphilus, Megasphaera*, *Finegoldia*), and Bacteroidetes (*Prevotella*).

### Comparison of bladder, gut, and vaginal isolates

To place the bladder microbiota in the context of other well-studied body sites, we compared our bladder genome collection with strains from 67 publicly available vaginal (Supplementary Table 2) and 120 gastrointestinal (Supplementary Table 3) species cultivated from unrelated healthy women. Based on whole-genome pairwise average nucleotide identity (ANI)^12–14^, only one species, *Bifidobacterium bifidum*, was detected in all three body sites (pairwise ANI > 95%, indicating same species). In contrast, 23 species were found in both the bladder and vaginal microbiota (ANI > 95%). We also identified seven species typically associated with UTIs (*E. cloacae*,* E. coli*,* P. aeruginosa*,* Bacillus infantis*,* K. pneumoniae*,* Gardnerella terrae*, and *Bacillus idriensis*), with three species isolated only from the bladder of symptomatic women (*P. aeruginosa*, *K. pneumoniae*, and *E. cloacae*) (Supplementary Table 4). Remarkably, from unrelated women, four species were identified (*Actinomyces neuii*, *Lactobacillus crispatus*,* L. gasseri*, and *L. jensenii*) that were highly similar between the bladder and vagina (Supplementary Table 5).

### Protein-coding functions of bladder isolates from asymptomatic individuals

**Fig. 2:**
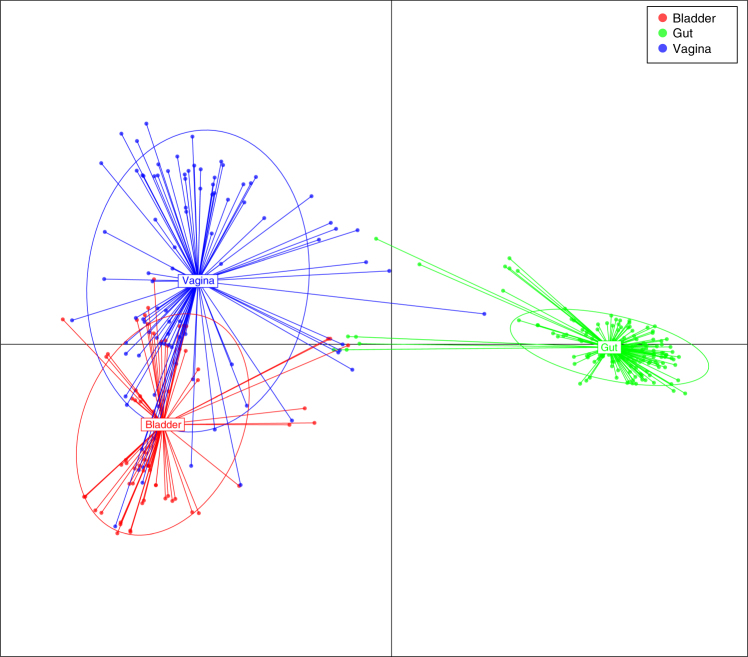
Functional diversity between genomes of bacterial strains isolated from the bladder, vagina, and intestine. Discriminant analysis of principal components using conserved protein domains (CDD). Each color dot represents a strain from 3 different niches: blue (vagina; *n* = 92), red (bladder; *n* = 67), and green (gut; *n* = 152)

To determine the protein functions encoded by the genomes of members of the healthy bladder microbiota and its relationship to the functions encoded by the genomes of microbiota from other body sites, we analyzed the genomes of bladder strains isolated from asymptomatic women, with gastrointestinal and vaginal strains isolated from other asymptomatic individuals. Applying conserved domain database (CDD)^15^ and discriminant analysis of principle components (DAPC)^16^, we compared the protein domains of the 67 bladder strains from healthy women with protein domains of existing 92 publicly available vaginal and 152 gastrointestinal strains cultivated from unrelated healthy individuals^17,18^. Consistent with the species analysis, this comparison demonstrates clear overlapping protein functions within the bladder and vaginal strains that were largely separated from protein functions found in the gastrointestinal strains (Fig. 2). Taken together, these results indicate the presence of shared functions across the bladder and vaginal microbiota that are clearly distinct from those of gastrointestinal species.

To understand the characteristics that differentiate vaginal and bladder bacteria from gastrointestinal tract bacteria, we next performed functional genomic comparisons. Applying Clusters of Orthologous Groups of proteins (COG) analysis, we identified significant enrichment of functional domains in the urogenital-associated bacteria associated with almost the entire mevalonate-dependent pathway for isoprenoid biosynthesis (Supplementary Table 6): 3-hydroxy-3-methylglutaryl CoA synthase (*q* < 1.33 × 10^−18^), 3-hydroxy-3-methylglutaryl CoA reductase (*q* < 5.17 × 10^−11^), mevalonate kinase (*q* < 5.84 × 10^−14^), and mevalonate pyrophosphate decarboxylase (*q* < 8.15 × 10^−15^). Also enriched was pantothenate kinase, a protein involved the biosynthesis of the coenzyme A required for isoprenoid biosnthesis (*q* < 1.1 × 10^−10^). Other enriched functional domains included several transport systems [the permease component of a predicted ABC-type exoprotein transport system (*q* < 5.71 × 10^−15^) and L-asparagine transporter and permeases (*q* < 1.40 × 10^−13^)] and a component of the Co/Zn/Cd efflux system (*q* < 2.53 × 10^−13^) involved in metal resistance. Also enriched were protoheme ferro-lyase (*q* < 4.40 × 10^−14^), the luciferase family of flavin-dependent oxidoreductases (*q* < 2.74 × 10^−13^), NAD(P)H-dependent FMN reductase (*q* < 6.52 × 10^−16^), a protein involved in ribonuclease reduction (*q* < 5.76 × 10^−16^), and the epsilon subunit of RNA polymerase, recently discovered in A/T-rich Gram-positive bacteria and thought to protect against phage infection (*q* < 5.71 × 10^−15^). In contrast, COG analysis identified significant enrichment of 5 functional domains associated with spore formation [YlmC (*q* < 1.67 × 10^−21^), SpoIIIAA (*q* < 2.61 × 10^−21^), CwlJ (*q* < 7.18 × 10^−20^), CotJC (*q* < 7.27 × 10^−^^19^), and SpmB (*q* < 5.31 × 10^−16^)], as well as iron-dependent oxidoreductases (*q* < 2.67 × 10^−^^17^) and aldo/keto oxidoreductases (*q* < 7.85 × 10^−21^) in the gastrointestinal tract (Supplementary Table 7). These results suggest spore formation and oxygen survival, while critical for transmission of gastrointestinal microbiota^19^, are significantly underrepresented in bacteria of the urogenital tract.

Functional enrichment of key metabolic pathways suggests specific nutritional selection on bacteria of the urogenital environment absent in bacteria of the gastrointestinal tract. Isoprenoids are essential to life, playing indispensable roles in membrane and peptidoglycan biosynthesis and electron transport. The vast majority of bacteria use the methylerythritol phosphate (MEP) pathway for isoprenoid biosynthesis. Intriguingly, this pathway concludes with two Fe-S cluster enzymes^20^. In contrast, the less common mevalonic acid (MVA) pathway, which is enriched in urogenital bacteria *Actinomyces neuii*,* Lactobacillus crispatus*,* L. gasseri*, and *L. jensenii*, contains no Fe-S clusters. Thus the enrichment of the MVA pathway over the more common MEP pathway may relate to iron availability within the vagina and bladder.

### Comparison of vaginal and bladder strains within individual women

Given the significant taxonomic and functional overlap of vaginal and bladder strains in asymptomatic women, we hypothesized the existence of interconnected urogenital microbiota, which we then sought to assess by culturing bacterial strains from the vaginal and bladder microbiota within an independent cohort of women with urgency urinary incontinence symptoms but no clinically detectable urinary infections. Four women contained a species in both anatomical sites (Supplementary Table 8), including *S. anginsosus*, which is associated with urgency urinary incontinence^2^, a putative non-pathogenic *E. coli*, and *L. iners* and *L. crispatus*. Both of these *Lactobacillus* species are associated with health in the vagina^21^, with the latter also associated with the bladder of asymptomatic women^2^.

**Fig. 3:**
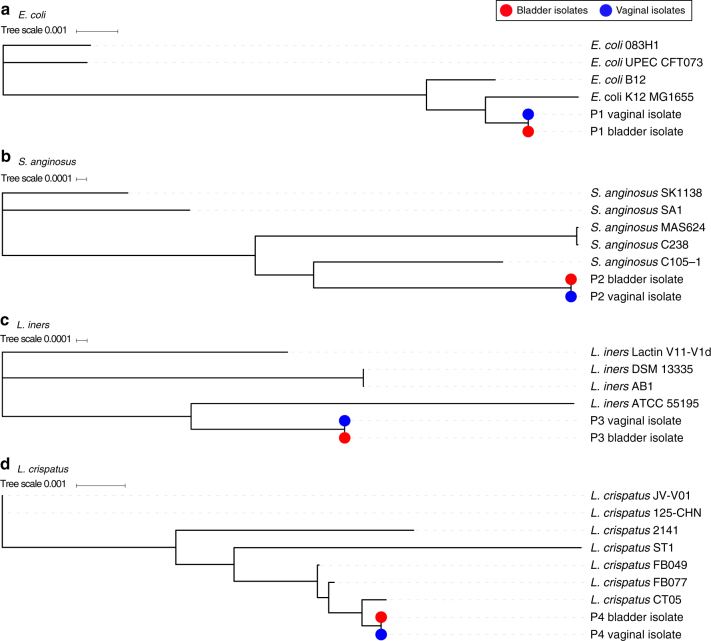
Phylogenetic comparison of bladder and vaginal strains isolated from individual women using the 40 universal core genes. **a** Maximum likelihood tree constructed from *E. coli* strains isolated from the bladder (red) and vagina (blue) of individual patient (P1) and four reference strains. **b** Maximum likelihood tree constructed from *S. anginosus* strains isolated from the bladder (red) and vagina (blue) of individual patient (P2) and five reference strains. **c** Maximum likelihood tree constructed from *L. iners* strains isolated from the bladder (red) and vagina (blue) of individual patient (P3) and four reference strains. **d** Maximum likelihood tree constructed from *L. crispatus* strains isolated from the bladder (red) and vagina (blue) of individual patient (P4) and seven reference strains

To determine whether these shared species belong to the same or distantly related bacterial lineages, we next sequenced and compared their genomes to each other and to the genomes of publicly available reference strains (Supplementary Table 9), using the 40-marker gene analysis^22,23^ and ANI analysis. In all the four strain sets tested, the vaginal strain was highly similar to the bladder strain (Fig. 3, blue and red dots). One individual contained *E. coli* strains that were 99.72% similar by ANI at both sites. These strains, which occurred in the absence of a clinically diagnosed urinary infection, were most closely related to known commensal strains and lacked both a Type III secretion system and other previously characterized pathogen-associated genes^24^ (Fig. 3a, Supplementary Table 10). The emerging uropathogen *S. anginosus* showed 99.77% similarity between strains from each site (Fig. 3b, Supplementary Table 11). Finally, the commensal *L. iners* and health-associated *L. crispatus* strains shared 99.99% (Fig. 3c, Supplementary Table 12) and 99.80% (Fig. 3d, Supplementary Table 13) similarity between the vagina and bladder. The existence of these closely related isolates provides strong evidence that bacterial movement between the vaginal and bladder microbiota is not only limited to ascending uropathogenic species, such as *E. coli*, as described previously^25^, but also includes health-associated commensal bacteria.

## Discussion

A growing body of work using catheterized urine in women has found associations between bladder microbiota composition and urgency urinary symptoms, response to anticholinergic medication^9^, risk of postoperative^11,26^ and post-instrumentation UTIs^8^, and kidney stones^27^. Here we provide an extensive, genome-sequenced culture collection representing approximately two thirds of bacterial strains detected in the sampled female bladders during both health and disease. Direct cultivation paired with whole-genome sequencing provides the ability to move beyond broad phylogenetic relatedness to perform strain-level tracking and functional analysis of these microbes and communities. The creation of this comprehensive reference collection, capturing the complete phylogenetic diversity currently cultivated from the bladder, represents a valuable resource to explore the function of both pathogenic and commensal bladder bacteria.

We have demonstrated for the first time that, like some pathogens, highly similar strains of health-associated commensal bacteria are found in both the bladder and vagina of the same individual. Previously, it was thought that a healthy vaginal microbiota was the major factor in preventing ascending infections from migrating into a sterile bladder. The data presented herein suggests that microbial sharing between the vaginal and bladder microbiota is not limited to known and emerging uropathogens, such as *E. coli* and *S. anginosus*, but also includes health-associated commensal bacteria, such as *L. iners* and *L. crispatus*. Now, we propose that some bacteria that can reside in both the bladder and vagina could provide protection against urinary infection, suggesting that the microbes of these adjacent pelvic floor niches could be considered to be a single urogenital microbiota. This insight, combined with this unique genome-sequenced culture collection, should alter the way we view the bacteria of the female pelvic floor both by enabling further research and by providing new diagnostic and treatment options for UTIs, urgency urinary incontinence, and other associated urinary tract disorders.

## Methods

### Patient recruitment

Following Loyola University Medical Center (LUMC) Institutional Review Board approval, participants gave verbal and written consent for chart abstraction and urine collection with analysis for research purposes. Recruitment and urine collection was performed by members of the Loyola Urinary Education and Research Collaborative who are part of the clinical practice of the Female Pelvic Medicine and Reconstructive Surgery Center at LUMC. Exclusion criteria for both cohorts included current UTI (based on urine dipstick) or history of recurrent UTI, antibiotic exposure in the past 4 weeks for any reason, immunologic deficiency, neurological disease known to affect the lower urinary tract, pelvic malignancy or radiation, untreated symptomatic pelvic organ prolapse (POP) greater than POP-Q stage II (vaginal protrusion >1 cm outside of the vaginal hymen), or pregnancy.

Patients were recruited as part of separate studies^2,9,27–29^. In the current study, representative isolates were selected for whole-genome sequencing in order to build as phylogenetically complete a dataset as possible. For this reason, only a few isolates from some patients have been included. For complete understanding of each patient’s microbiome or for additional metadata, please refer to the primary publications listed in Supplementary Data 1.

### Urine collection and EQUC bacterial culturing

Urine was collected aseptically via TUC and was placed in a BD Vacutainer Plus C&S preservative tube for culturing. Previous work has shown that the microbiota detected in suprapubic aspirates are indistinguishable from the microbiota detected in urine obtain by TUC but distinct from the microbiota in voided urine and vaginal swabs^1^. Thus aspiration and catheterization sample the same niche. Since aspiration bypasses the vulva, vagina, and urethra, this niche must be the bladder. Since it is less invasive than aspiration, TUC is the urine sampling method of choice. We note that because TUC is a transient procedure lasting no more than a few seconds, it is not associated with CAUTI. This study does not use nor address indwelling catheters that, due to biofilm formation, are associated with CAUTIs.

Four patients were chosen to sample both the bladder and vaginal environments (Fig. 3). For these women, the vaginal swab was collected from the posterior fornix prior to the catheterized urine collection.

All samples underwent standard urine culture (SUC) as well as EQUC. For SUC, 1 µl of urine was inoculated onto 5% sheep blood agar plate (BAP) and MacConkey agar plate (BD BBL prepared plated media), incubated aerobically at 35 °C for 24 h. The detection level was 1000 CFU/ml, represented by 1 colony of growth on either plate. If no growth was observed, the culture was reported as “no growth”, indicating no growth of bacteria at the lowest dilution, i.e., 1:1000. SUC results are listed in Supplementary Data 1.

EQUC was performed as described previously^3^. Briefly, 100 µl of urine was grown under five conditions with BD BBL prepared plated media: (1) BAP in CO_2_ for 48 h, (2) chocolate agar (CHOC) in CO_2_ for 48 h, (3) colistin and nalidixic acid (CNA) agar in CO_2_ for 48 h, (4) CDC anaerobe BAP in an anaerobic jar for 48 h, and (5) BAP in aerobic conditions (BD GasPak Anaerobe Sachets) for 48 h. The detection level was 10 CFU/ml, represented by 1 colony of growth on any of the plates. EQUC results are listed in Supplementary Data 1.

Vaginal swabs were collected using BD Liquid Amies Elution Swab (ESwab) collection system. To compare vagina and bladder culture data (Fig. 3, Supplementary Table 9), vaginal samples were cultured using a modified EQUC protocol, with 10 µl of urine plated onto BAP and CNA grown for 48 h in 5% CO_2_, and anaerobic BAP grown for 48 h under anaerobic conditions (BD BBL prepared plated media and BD GasPak Anaerobe Sachets).

Each morphologically distinct colony type was isolated on a different plate of the same medium to prepare a pure culture that was used for identification. Matrix-assisted laser desorption ionization–time of flight mass spectrophotometry with the MALDI Biotyper 3.0 software program (Bruker Daltonics, Billerica, MA) was used to identify the bacterial strains, as described elsewhere^3^.

The 149 bacterial strains are available upon request.

### Genome sequencing and annotation

The isolates were grown in their preferred medium and pelleted. Genomic DNA was extracted from pelleted cells using a phenol–chloroform method^30^. DNA was prepared and sequenced using the Illumina Hi-Seq platform with library fragment sizes of 200–300 bp and a read length of 100 bp at the Wellcome Sanger Institute, as previously described^31^. Annotated assemblies were produced using the pipeline described previously^32^. For each sample, sequence reads were used to create multiple assemblies using Velvet v1.2^33^ and VelvetOptimiser v2.2.5 (https://github.com/tseemann/VelvetOptimiser). An assembly improvement step was applied to the assembly with the best N50 and contigs were scaffolded using SSPACE^34^ and sequence gaps filled using GapFiller^35^. Automated annotation was performed using PROKKA v1.11^36^.

### Whole-genome metagenomic analysis

Whole-genome metagenomic sequencing was performed on the Illumina HiSeq 2500 as described previously^18^ with human contaminating reads filtered by mapping to the Human reference genome (hg19) with bowtie2^37^. Filtered sequences were compared at the genus and species levels using lowest common ancestor analysis previously described^38^ and by relative abundance at the sequence level by alignment using the bowtie2 algorithm to the complete bladder culture collection genome catalog.

### Phylogenetic analysis and average nucleotide identity analysis

The phylogenetic analysis was conducted by extracting amino acid sequence of 40 universal single copy marker genes^22,23^ from bacterial collection using SpecI^39^. The protein sequences were concatenated and aligned with MAFFT v. 7.20^40^, and maximum-likelihood trees were constructed using FastTree^41^ with default settings. All phylogenetic trees were visualized in iTOL^42^. ANI was calculated by performing pairwise comparison of genome assemblies using MUMmer^43^.

### Functional genomic analysis

To identify protein domains in a genome, we performed RPS-BLAST using CDD^44^. All protein domains were classified in different functional categories using COG database^45^ and were used to perform DAPC^16^ implemented in the R package Adegenet v2.0.1^46^. Domain enrichment was calculated using one-sided Fisher’s exact test with *p*-value adjusted by Hochberg method in R v3.2.2.

### Data availability

Genome and metagenome sequences have been deposited in the European Nucleotide Archive. Accession codes for genome sequences are listed in Supplementary Data 1 and all supplementary tables. Metagenomic sequences are listed in Supplementary Table 1. Other relevant data supporting the findings of the study are available in this article and its Supplementary Information files or from the corresponding authors upon request.

## Electronic supplementary material

Supplementary Information

Description of Additional Supplementary Files

Supplementary Data 1
