# Supplementary material for: Culturing of female bladder bacteria reveals an interconnected urogenital microbiota
Source: Nat Commun. 2018 Apr 19;9:1557. doi: 10.1038/s41467-018-03968-5 (PMC5908796; doi:10.1038/s41467-018-03968-5)
Supplement: Supplementary file 1 — Supplementary Information [file 41467_2018_3968_MOESM1_ESM.pdf]

Culturing of female bladder bacteria reveals an interconnected urogenital microbiota

Thomas-White et al.

Supplementary Material

Supplementary Table 1: List of the metagenomic samples and the relative coverage of the bladder culture collection

ENA* Identifier	Coverage by Abundance (%)	Coverage by Genera (Total)	Coverage by Genera (%)
ERS685380 [https://www.ebi.ac.uk/ena/data/view/ERS685380]	63.9	6 (10)	60.0
ERS685386 [https://www.ebi.ac.uk/ena/data/view/ERS685386]	62.8	6 (8)	75.0
ERS685396 [https://www.ebi.ac.uk/ena/data/view/ERS685396]	65.9	7 (12)	58.3
ERS685400 [https://www.ebi.ac.uk/ena/data/view/ERS685400]	98.2	2 (2)	100.0
ERS685390 [https://www.ebi.ac.uk/ena/data/view/ERS685390]	97.1	3 (3)	100.0
ERS685384 [https://www.ebi.ac.uk/ena/data/view/ERS685384]	72.4	6 (9)	66.7
ERS685395 [https://www.ebi.ac.uk/ena/data/view/ERS685395]	89.3	2 (2)	100.0
ERS685397 [https://www.ebi.ac.uk/ena/data/view/ERS685397]	67.1	11 (17)	64.7
ERS685401 [https://www.ebi.ac.uk/ena/data/view/ERS685401]	40.9	9 (15)	60.0
ERS685403 [https://www.ebi.ac.uk/ena/data/view/ERS685403]	34.9	9 (12)	75.0
ERS685382 [https://www.ebi.ac.uk/ena/data/view/ERS685382]	44.5	7 (17)	41.2
ERS685385 [https://www.ebi.ac.uk/ena/data/view/ERS685385]	60.0	7 (11)	63.6

***European Nucleotide Archive**

Supplementary Table 2: List of the publicly available genomes of vaginal strains analysed in this study. These vaginal genomes are all publicly available, the majority of which were collected by the HMP[1]. Vaginal genomes were only included if the metadata specifically stated that the strain had been collected from the vagina ("urogenital" isolates were excluded).

Assembly Accession	Species
GCA_001546135.1 [https://www.ncbi.nlm.nih.gov/assembly/GCF_001546165.1/]	*Actinomyces neuii* MJR8396A
GCA_000758775.1 [https://www.ncbi.nlm.nih.gov/assembly/GCF_000758775.1/]	*Actinomyces* sp S4-C9
GCA_000758755.1 [https://www.ncbi.nlm.nih.gov/assembly/GCF_000758755.1/]	*Actinomyces* sp S6-Spd3
GCA_001552755.1 [https://www.ncbi.nlm.nih.gov/assembly/GCF_001552755.1/]	*Aerococcus christensenii* KA00635
GCA_000178435.1 [https://www.ncbi.nlm.nih.gov/assembly/GCF_000178435.1/]	*Aerococcus viridans* ATCC11563
GCA_001546145.1 [https://www.ncbi.nlm.nih.gov/assembly/GCF_001546145.1/]	*Alloscardovia omnicolens* CMW7705A
GCA_000758765.1 [https://www.ncbi.nlm.nih.gov/assembly/GCF_000758765.1/]	*Anaerococcus lactolyticus* S7-1-13
GCA_001546165.1 [https://www.ncbi.nlm.nih.gov/assembly/GCF_001546165.1/]	*Anaerococcus tetradius* MJR8151
GCA_000758825.1 [https://www.ncbi.nlm.nih.gov/assembly/GCF_000758825.1/]	*Arcanobacterium* sp S3PF19
GCA_001552785.1 [https://www.ncbi.nlm.nih.gov/assembly/GCF_001552785.1/]	*Atopobium deltae* LSCR 12804
GCA_000758945.1 [https://www.ncbi.nlm.nih.gov/assembly/GCF_000758945.1/]	*Atopobium parvulum* DNF00906
GCA_000159235.2 [https://www.ncbi.nlm.nih.gov/assembly/GCF_000159235.2/]	*Atopobium vaginae* DSM15829
GCA_001546215.1 [https://www.ncbi.nlm.nih.gov/assembly/GCF_001546215.1/]	*Bacillus coagulans* GED7749B
GCA_001552765.1 [https://www.ncbi.nlm.nih.gov/assembly/GCF_001552765.1/]	Bacteroidales bacterium KA00344
GCA_001546225.1 [https://www.ncbi.nlm.nih.gov/assembly/GCF_001546225.1/]	*Bifidobacterium bifidum* MJR8628B
GCA_001546235.1 [https://www.ncbi.nlm.nih.gov/assembly/GCF_001546235.1/]	*Bifidobacterium breve* GED8481
GCA_000466545.1 [https://www.ncbi.nlm.nih.gov/assembly/GCF_000466545.1/]	*Bifidobacterium breve* JCP7499
GCA_000146775.1 [https://www.ncbi.nlm.nih.gov/assembly/GCF_000146775.1/]	*Bifidobacterium dentium* ATCC27679
GCA_000759055.1 [https://www.ncbi.nlm.nih.gov/assembly/GCF_000759055.1/]	*Corynebacterium tuscaniense* DNF00037
GCA_001552885.1 [https://www.ncbi.nlm.nih.gov/assembly/GCF_001552885.1/]	Clostridiales bacterium KA00274
GCA_000758905.1 [https://www.ncbi.nlm.nih.gov/assembly/GCF_000758905.1/]	Clostridiales bacterium S5-A14a

GCA_001552965.1 [https://www.ncbi.nlm.nih.gov/assembly/GCF_001552965.1/]	*Corynebacterium* sp DNF00584
GCA_000194985.1 [https://www.ncbi.nlm.nih.gov/assembly/GCF_000194985.1/]	*Dialister micraerophilus* DSM19965
GCF_001546375.1 [https://www.ncbi.nlm.nih.gov/assembly/GCF_001546375.1/]	*Enterococcus faecium* MJR8396B
GCA_000159695.1 [https://www.ncbi.nlm.nih.gov/assembly/GCF_000159695.1/]	*Finegoldia magna* ATCC53516
GCA_000178895.1 [https://www.ncbi.nlm.nih.gov/assembly/GCF_000178895.1/]	*Fusobacterium nucleatum* ATCC23726
GCA_001546435.1 [https://www.ncbi.nlm.nih.gov/assembly/GCF_001546435.1/]	*Fusobacterium nucleatum* MJR7757B
GCA_001546445.1 [https://www.ncbi.nlm.nih.gov/assembly/GCF_001546445.1/]	*Gardnerella vaginalis* GED7275B
GCA_001546455.1 [https://www.ncbi.nlm.nih.gov/assembly/GCF_001546455.1/]	*Gardnerella vaginalis* GED7760B
GCA_001553005.1 [https://www.ncbi.nlm.nih.gov/assembly/GCF_001553005.1/]	*Gemella asaccharolytica* KA00071
GCA_001553035.1 [https://www.ncbi.nlm.nih.gov/assembly/GCF_001553035.1/]	*Gemella haemolysans* DNF01167
GCA_001552975.1 [https://www.ncbi.nlm.nih.gov/assembly/GCF_001552975.1/]	*Lachnoanaerobaculum saburreum* DNF00896
GCA_000177035.2 [https://www.ncbi.nlm.nih.gov/assembly/GCF_000177035.2/]	*Lactobacillus gasseri* SV-16A-US
GCA_000161915.2 [https://www.ncbi.nlm.nih.gov/assembly/GCF_000161915.2/]	*Lactobacillus crispatus* MV-1A-US
GCA_000176975.2 [https://www.ncbi.nlm.nih.gov/assembly/GCF_000176975.2/]	*Lactobacillus crispatus* SJ-3C-US
GCA_001541385.1 [https://www.ncbi.nlm.nih.gov/assembly/GCF_001541385.1/]	*Lactobacillus crispatus* VMC3
GCA_001541405.1 [https://www.ncbi.nlm.nih.gov/assembly/GCF_001541405.1/]	*Lactobacillus crispatus* VMC4
GCA_001541515.1 [https://www.ncbi.nlm.nih.gov/assembly/GCF_001541515.1/]	*Lactobacillus crispatus* VMC5
GCA_001541535.1 [https://www.ncbi.nlm.nih.gov/assembly/GCF_001541535.1/]	*Lactobacillus crispatus* VMC7
GCA_001541585.1 [https://www.ncbi.nlm.nih.gov/assembly/GCF_001541585.1/]	*Lactobacillus crispatus* VMC8
GCA_000175055.1 [https://www.ncbi.nlm.nih.gov/assembly/GCF_000175055.1/]	*Lactobacillus gasseri* 202-4
GCA_000176995.2 [https://www.ncbi.nlm.nih.gov/assembly/GCF_000176995.2/]	*Lactobacillus gasseri* SJ-9E-US
GCA_000185405.1 [https://www.ncbi.nlm.nih.gov/assembly/GCF_000185405.1/]	*Lactobacillus iners* ATCC55195
GCA_000179955.1 [https://www.ncbi.nlm.nih.gov/assembly/GCF_000179955.1/]	*Lactobacillus iners* LEAF 2052A-d
GCA_000149145.2 [https://www.ncbi.nlm.nih.gov/assembly/GCF_000149145.2/]	*Lactobacillus iners* SPIN 2503V10-d

GCA_000162435.1 [https://www.ncbi.nlm.nih.gov/assembly/GCF_000162435.1/]	*Lactobacillus jensenii* 115-3-CHN
GCA_000162335.1 [https://www.ncbi.nlm.nih.gov/assembly/GCF_000162335.1/]	*Lactobacillus jensenii* SJ-7A-US
GCA_000175035.1 [https://www.ncbi.nlm.nih.gov/assembly/GCF_000175035.1/]	*Lactobacillus jensenii* 269-3
GCA_000180015.1 [https://www.ncbi.nlm.nih.gov/assembly/GCF_000180015.1/]	*Lactobacillus oris* PB013-T2-3
GCA_000699985.1 [https://www.ncbi.nlm.nih.gov/assembly/GCF_000699985.1/]	*Lactobacillus rhamnosus* 51B
GCA_000179475.1 [https://www.ncbi.nlm.nih.gov/assembly/GCF_000179475.1/]	*Lactobacillus salivarius* ACS-116-V-Col5a
GCA_000159435.1 [https://www.ncbi.nlm.nih.gov/assembly/GCF_000159435.1/]	*Lactobacillus vaginalis* ATCC49540
GCA_001546855.1 [https://www.ncbi.nlm.nih.gov/assembly/GCF_001546855.1/]	*Megasphaera* sp MJR8396C
GCA_000146285.1 [https://www.ncbi.nlm.nih.gov/assembly/GCF_000146285.1/]	*Mobiluncus curtisii* Subsp curtisii ATCC35241
GCA_000185445.1 [https://www.ncbi.nlm.nih.gov/assembly/GCF_000185445.1/]	*Mobiluncus curtisii* Subsp holmesii ATCC35242
GCA_000146895.1 [https://www.ncbi.nlm.nih.gov/assembly/GCF_000146895.1/]	*Mobiluncus mulieris* ATCC35239
GCA_000164135.1 [https://www.ncbi.nlm.nih.gov/assembly/GCF_000164135.1/]	*Mycobacterium parascrofulaceum* BAA614
GCA_001553055.1 [https://www.ncbi.nlm.nih.gov/assembly/GCF_001553055.1/]	*Olsenella* sp DNF00959
GCA_001553085.1 [https://www.ncbi.nlm.nih.gov/assembly/GCF_001553085.1/]	*Parvimonas* sp KA00067
GCA_001553115.1 [https://www.ncbi.nlm.nih.gov/assembly/GCF_001553115.1/]	*Peptoniphilus* coxii DNF00729
GCA_000146345.1 [https://www.ncbi.nlm.nih.gov/assembly/GCF_000146345.1/]	*Peptoniphilus* duerdenii BAA-1640
GCA_001546555.1 [https://www.ncbi.nlm.nih.gov/assembly/GCF_001546555.1/]	*Peptoniphilus* harei CMW7756A
GCA_001553125.1 [https://www.ncbi.nlm.nih.gov/assembly/GCF_001553125.1/]	*Peptoniphilus* sp DNF00840
GCA_001553145.1 [https://www.ncbi.nlm.nih.gov/assembly/GCF_001553145.1/]	*Peptostreptococcus anaerobius* KA00810
GCA_000758885.1 [https://www.ncbi.nlm.nih.gov/assembly/GCF_000758885.1/]	*Peptostreptococcus* sp MV1
GCA_000759315.1 [https://www.ncbi.nlm.nih.gov/assembly/GCF_000759315.1/]	*Prevotella amnii* DNF00058
GCA_001553225.1 [https://www.ncbi.nlm.nih.gov/assembly/GCF_001553225.1/]	*Prevotella amnii* DNF00307
GCA_000759045.1 [https://www.ncbi.nlm.nih.gov/assembly/GCF_000759045.1/]	*Prevotella bivia* DNF00188
GCA_000759245.1 [https://www.ncbi.nlm.nih.gov/assembly/GCF_000759245.1/]	*Prevotella bivia* DNF00320

GCA_000759165.1 [https://www.ncbi.nlm.nih.gov/assembly/GCF_000759165.1/]	*Prevotella bivia* DNF00650
GCA_001546565.2 [https://www.ncbi.nlm.nih.gov/assembly/GCF_001546565.2/]	*Prevotella bivia* GED7760C
GCA_001546595.1 [https://www.ncbi.nlm.nih.gov/assembly/GCF_001546595.1/]	*Prevotella corporis* MJR7716
GCA_000759205.1 [https://www.ncbi.nlm.nih.gov/assembly/GCF_000759205.1/]	*Prevotella denticola* DNF00960
GCA_000759225.1 [https://www.ncbi.nlm.nih.gov/assembly/GCF_000759225.1/]	*Prevotella disiens* DNF00882
GCA_000762405.1 [https://www.ncbi.nlm.nih.gov/assembly/GCF_000762405.1/]	*Prevotella timonensis* S9-PR14
GCA_001546875.1 [https://www.ncbi.nlm.nih.gov/assembly/GCF_001546875.1/]	*Propionibacterium avidum* MJR7694
GCA_001553525.1 [https://www.ncbi.nlm.nih.gov/assembly/GCF_001553525.1/]	*Proteus mirabilis* GED7834
GCA_000759125.1 [https://www.ncbi.nlm.nih.gov/assembly/GCF_000759125.1/]	*Staphylococcus haemolyticus* DNF00585
GCA_001546615.1 [https://www.ncbi.nlm.nih.gov/assembly/GCF_001546615.1/]	*Staphylococcus lugdunensis* MJR7738
GCA_001546635.1 [https://www.ncbi.nlm.nih.gov/assembly/GCF_001546635.1/]	*Staphylococcus simulans* MJR7712
GCA_000146405.1 [https://www.ncbi.nlm.nih.gov/assembly/GCF_000146405.1/]	*Streptococcus equinus* ATCC700338
GCA_001546795.1 [https://www.ncbi.nlm.nih.gov/assembly/GCF_001546795.1/]	*Streptococcus mitis* CMW7705B
GCA_001552895.1 [https://www.ncbi.nlm.nih.gov/assembly/GCF_001552895.1/]	*Tissierella bacterium* KA00581
GCA_000758845.1 [https://www.ncbi.nlm.nih.gov/assembly/GCF_000758845.1/]	*Tissierella bacterium* S7-1-4
GCA_001553285.1 [https://www.ncbi.nlm.nih.gov/assembly/GCF_001553285.1/]	*Varibaculum cambriense* DNF00696
GCA_001553315.1 [https://www.ncbi.nlm.nih.gov/assembly/GCF_001553315.1/]	*Veillonella dispar* DNF00926
GCA_000759285.1 [https://www.ncbi.nlm.nih.gov/assembly/GCF_000759285.1/]	*Veillonella montpellierensis* DNF00314
GCA_000215025.2 [https://www.ncbi.nlm.nih.gov/assembly/GCF_000215025.2/]	*Veillonella parvula* ACS-068-V-Sch12
GCA_001553345.1 [https://www.ncbi.nlm.nih.gov/assembly/GCF_001553345.1/]	*Veillonella* sp DNF00869
GCA_001553355.1 [https://www.ncbi.nlm.nih.gov/assembly/GCF_001553355.1/]	Veillonellaceae bacterium DNF00626
GCA_001553395.1 [https://www.ncbi.nlm.nih.gov/assembly/GCF_001553395.1/]	Veillonellaceae bacterium DNF00751
GCA_001553405.1 [https://www.ncbi.nlm.nih.gov/assembly/GCF_001553405.1/]	Veillonellaceae bacterium KA00182

Supplementary Table 3: List of genomes from gastrointestinal strains analysed. These strains were originally collected in a previous study[2].

ENA* id	Species
ERR1022418 [https://www.ebi.ac.uk/ena/data/view/ERR1022418]	*Intestinibacter bartlettii*
ERR1022321 [https://www.ebi.ac.uk/ena/data/view/ERR1022321]	*Roseburia faecis*
ERR1022435 [https://www.ebi.ac.uk/ena/data/view/ERR1022435]	*Blautia* nov.
ERR1022358 [https://www.ebi.ac.uk/ena/data/view/ERR1022358]	*Bacteroides vulgatus*
ERR1022308 [https://www.ebi.ac.uk/ena/data/view/ERR1022308]	Lachnospiraceae nov.
ERR1022387 [https://www.ebi.ac.uk/ena/data/view/ERR1022387]	*Lachnoclostridium* nov.
ERR1022421 [https://www.ebi.ac.uk/ena/data/view/ERR1022421]	*Sarcina* nov.
ERR1022291 [https://www.ebi.ac.uk/ena/data/view/ERR1022291]	*Ruminococcus torques*
ERR1022428 [https://www.ebi.ac.uk/ena/data/view/ERR1022428]	*Dorea longicatena*
ERR1022370 [https://www.ebi.ac.uk/ena/data/view/ERR1022370]	*Megasphaera* nov.
ERR1022360 [https://www.ebi.ac.uk/ena/data/view/ERR1022360]	*Bacteroides uniformis*
ERR1022433 [https://www.ebi.ac.uk/ena/data/view/ERR1022433]	Lachnospiraceae nov.
ERR1022323 [https://www.ebi.ac.uk/ena/data/view/ERR1022323]	*Turicibacter sanguinis*
ERR1022329 [https://www.ebi.ac.uk/ena/data/view/ERR1022329]	*Bacteroides salyersiae*
ERR1022279 [https://www.ebi.ac.uk/ena/data/view/ERR1022279]	*Faecalibacterium prausnitzii*
ERR1022288 [https://www.ebi.ac.uk/ena/data/view/ERR1022288]	*Anaerostipes hadrus*
ERR1022305 [https://www.ebi.ac.uk/ena/data/view/ERR1022305]	*Lachnoclostridium* nov.
ERR1022450 [https://www.ebi.ac.uk/ena/data/view/ERR1022450]	*Intestinimonas butyriciproducens*
ERR1022307 [https://www.ebi.ac.uk/ena/data/view/ERR1022307]	*Fusicatenibacter saccharivorans*
ERR1022345 [https://www.ebi.ac.uk/ena/data/view/ERR1022345]	*Roseburia inulinivorans*
ERR1022281 [https://www.ebi.ac.uk/ena/data/view/ERR1022281]	*Parabacteroides distasonis*
ERR1022299 [https://www.ebi.ac.uk/ena/data/view/ERR1022299]	Ruminococcaceae nov.

ERR1022335 [https://www.ebi.ac.uk/ena/data/view/ERR1022335]	*Bifidobacterium bifidum*
ERR1022408 [https://www.ebi.ac.uk/ena/data/view/ERR1022408]	*Ruminococcus bicirculans*
ERR1022460 [https://www.ebi.ac.uk/ena/data/view/ERR1022460]	*Bacteroides caccae*
ERR1022381 [https://www.ebi.ac.uk/ena/data/view/ERR1022381]	Lachnospiraceae nov.
ERR1022320 [https://www.ebi.ac.uk/ena/data/view/ERR1022320]	*Bifidobacterium adolescentis*
ERR1022452 [https://www.ebi.ac.uk/ena/data/view/ERR1022452]	Ruminococcaceae nov.
ERR1022474 [https://www.ebi.ac.uk/ena/data/view/ERR1022474]	*Roseburia intestinalis*
ERR1022432 [https://www.ebi.ac.uk/ena/data/view/ERR1022432]	*Ruminococcus faecis*
ERR1022275 [https://www.ebi.ac.uk/ena/data/view/ERR1022275]	*Blautia* nov.
ERR1022422 [https://www.ebi.ac.uk/ena/data/view/ERR1022422]	*Anaerostipes hadrus*
ERR1022313 [https://www.ebi.ac.uk/ena/data/view/ERR1022313]	Ruminococcaceae nov.
ERR1022457 [https://www.ebi.ac.uk/ena/data/view/ERR1022457]	*Bacteroides fragilis*
ERR1022414 [https://www.ebi.ac.uk/ena/data/view/ERR1022414]	*Bacteroides cellulosilyticus*
ERR1022352 [https://www.ebi.ac.uk/ena/data/view/ERR1022352]	*Lachnospira pectinoschiza*
ERR1022391 [https://www.ebi.ac.uk/ena/data/view/ERR1022391]	*Lachnospira* nov.
ERR1022289 [https://www.ebi.ac.uk/ena/data/view/ERR1022289]	Lachnospiraceae nov.
ERR1022319 [https://www.ebi.ac.uk/ena/data/view/ERR1022319]	*Bacteroides finegoldii*
ERR1022325 [https://www.ebi.ac.uk/ena/data/view/ERR1022325]	*Ruminococcus torques*
ERR1022304 [https://www.ebi.ac.uk/ena/data/view/ERR1022304]	*Ruminococcus torques*
ERR1022274 [https://www.ebi.ac.uk/ena/data/view/ERR1022274]	Lachnospiraceae nov.
ERR1022347 [https://www.ebi.ac.uk/ena/data/view/ERR1022347]	*Sarcina* nov.
ERR1022478 [https://www.ebi.ac.uk/ena/data/view/ERR1022478]	Lachnospiraceae nov.
ERR1022394 [https://www.ebi.ac.uk/ena/data/view/ERR1022394]	*Lachnospira* nov.
ERR1022280 [https://www.ebi.ac.uk/ena/data/view/ERR1022280]	*Turicibacter sanguinis*

ERR1022424 [https://www.ebi.ac.uk/ena/data/view/ERR1022424]	*Blautia* nov.
ERR1022353 [https://www.ebi.ac.uk/ena/data/view/ERR1022353]	Lachnospiraceae nov.
ERR1022405 [https://www.ebi.ac.uk/ena/data/view/ERR1022405]	*Lachnoclostridium* nov.
ERR1022437 [https://www.ebi.ac.uk/ena/data/view/ERR1022437]	*Fusicatenibacter saccharivorans*
ERR1022466 [https://www.ebi.ac.uk/ena/data/view/ERR1022466]	*Romboutsia* nov.
ERR1022276 [https://www.ebi.ac.uk/ena/data/view/ERR1022276]	*Lachnoclostridium* nov.
ERR1022480 [https://www.ebi.ac.uk/ena/data/view/ERR1022480]	Lachnospiraceae nov.
ERR1022458 [https://www.ebi.ac.uk/ena/data/view/ERR1022458]	*Bacteroides vulgatus*
ERR1022378 [https://www.ebi.ac.uk/ena/data/view/ERR1022378]	*Blautia* nov.
ERR1022464 [https://www.ebi.ac.uk/ena/data/view/ERR1022464]	Erysipelotrichaceae nov.
ERR1022380 [https://www.ebi.ac.uk/ena/data/view/ERR1022380]	Lachnospiraceae nov.
ERR1022339 [https://www.ebi.ac.uk/ena/data/view/ERR1022339]	*Blautia* nov.
ERR1022382 [https://www.ebi.ac.uk/ena/data/view/ERR1022382]	*Coprococcus comes*
ERR1022349 [https://www.ebi.ac.uk/ena/data/view/ERR1022349]	*Eubacterium ramulus*
ERR1022332 [https://www.ebi.ac.uk/ena/data/view/ERR1022332]	Erysipelotrichaceae nov.
ERR1022399 [https://www.ebi.ac.uk/ena/data/view/ERR1022399]	*Eubacterium* rectale
ERR1022361 [https://www.ebi.ac.uk/ena/data/view/ERR1022361]	*Bacteroides* nov.
ERR1022351 [https://www.ebi.ac.uk/ena/data/view/ERR1022351]	Lachnospiraceae nov.
ERR1022365 [https://www.ebi.ac.uk/ena/data/view/ERR1022365]	*Enorma massiliensis*
ERR1022309 [https://www.ebi.ac.uk/ena/data/view/ERR1022309]	*Dorea longicatena*
ERR1022455 [https://www.ebi.ac.uk/ena/data/view/ERR1022455]	Christensenellaceae nov.
ERR1022282 [https://www.ebi.ac.uk/ena/data/view/ERR1022282]	*Collinsella aerofaciens*
ERR1022371 [https://www.ebi.ac.uk/ena/data/view/ERR1022371]	*Sarcina* nov.
ERR1022416 [https://www.ebi.ac.uk/ena/data/view/ERR1022416]	*Collinsella aerofaciens*

ERR1022412 [https://www.ebi.ac.uk/ena/data/view/ERR1022412]	*Bacteroides uniformis*
ERR1022316 [https://www.ebi.ac.uk/ena/data/view/ERR1022316]	*Sarcina* nov.
ERR1022295 [https://www.ebi.ac.uk/ena/data/view/ERR1022295]	*Blautia* nov.
ERR1022395 [https://www.ebi.ac.uk/ena/data/view/ERR1022395]	*Intestinibacter bartlettii*
ERR1022369 [https://www.ebi.ac.uk/ena/data/view/ERR1022369]	Erysipelotrichaceae nov.
ERR1022294 [https://www.ebi.ac.uk/ena/data/view/ERR1022294]	*Blautia* nov.
ERR1022411 [https://www.ebi.ac.uk/ena/data/view/ERR1022411]	*Bacteroides vulgatus*
ERR1022434 [https://www.ebi.ac.uk/ena/data/view/ERR1022434]	Lachnoclostridium nov.
ERR1022392 [https://www.ebi.ac.uk/ena/data/view/ERR1022392]	Lachnoclostridium nov.
ERR1022333 [https://www.ebi.ac.uk/ena/data/view/ERR1022333]	Erysipelotrichaceae nov.
ERR1022413 [https://www.ebi.ac.uk/ena/data/view/ERR1022413]	*Bacteroides thetaiotaomicron*
ERR1022463 [https://www.ebi.ac.uk/ena/data/view/ERR1022463]	*Turicibacter sanguinis*
ERR1022315 [https://www.ebi.ac.uk/ena/data/view/ERR1022315]	*Blautia hydrogenotrophica*
ERS417308 [https://www.ebi.ac.uk/ena/data/view/ERS417308]	*Bifidobacterium adolescentis*
ERR1022471 [https://www.ebi.ac.uk/ena/data/view/ERR1022471]	*Blautia luti*
ERR1022363 [https://www.ebi.ac.uk/ena/data/view/ERR1022363]	*Bacteroides uniformis*
ERR1022454 [https://www.ebi.ac.uk/ena/data/view/ERR1022454]	*Intestinimonas* nov.
ERR1022400 [https://www.ebi.ac.uk/ena/data/view/ERR1022400]	*Fusicatenibacter saccharivorans*
ERR1022453 [https://www.ebi.ac.uk/ena/data/view/ERR1022453]	*Anaerotruncus colihominis*
ERR1022465 [https://www.ebi.ac.uk/ena/data/view/ERR1022465]	*Romboutsia* nov.
ERR1022439 [https://www.ebi.ac.uk/ena/data/view/ERR1022439]	*Eisenbergiella tayi*
ERR1022423 [https://www.ebi.ac.uk/ena/data/view/ERR1022423]	*Anaerostipes hadrus*
ERR1022481 [https://www.ebi.ac.uk/ena/data/view/ERR1022481]	Lachnospiraceae nov.
ERR1022272 [https://www.ebi.ac.uk/ena/data/view/ERR1022272]	Lachnospiraceae nov.

ERR1022286 [https://www.ebi.ac.uk/ena/data/view/ERR1022286]	*Mitsuokella jalaludinii*
ERR1022300 [https://www.ebi.ac.uk/ena/data/view/ERR1022300]	*Collinsella aerofaciens*
ERR1022342 [https://www.ebi.ac.uk/ena/data/view/ERR1022342]	Lachnospiraceae nov.
ERR1022398 [https://www.ebi.ac.uk/ena/data/view/ERR1022398]	Lachnospiraceae nov.
ERR1022443 [https://www.ebi.ac.uk/ena/data/view/ERR1022443]	Ruminococcaceae nov.
ERR1022431 [https://www.ebi.ac.uk/ena/data/view/ERR1022431]	Lachnospiraceae nov.
ERR1022456 [https://www.ebi.ac.uk/ena/data/view/ERR1022456]	*Bacteroides* nov.
ERR1022440 [https://www.ebi.ac.uk/ena/data/view/ERR1022440]	*Ruminiclostridium* nov.
ERR1022318 [https://www.ebi.ac.uk/ena/data/view/ERR1022318]	*Eubacterium rectale*
ERR1022451 [https://www.ebi.ac.uk/ena/data/view/ERR1022451]	Ruminococcaceae nov.
ERR1022311 [https://www.ebi.ac.uk/ena/data/view/ERR1022311]	Ruminococcaceae nov.
ERR1022445 [https://www.ebi.ac.uk/ena/data/view/ERR1022445]	Ruminococcaceae nov.
ERR1022426 [https://www.ebi.ac.uk/ena/data/view/ERR1022426]	*Blautia* nov.
ERR1022344 [https://www.ebi.ac.uk/ena/data/view/ERR1022344]	*Roseburia* nov.
ERR1022334 [https://www.ebi.ac.uk/ena/data/view/ERR1022334]	Clostridiales incertae sedis nov.
ERR1022430 [https://www.ebi.ac.uk/ena/data/view/ERR1022430]	*Lachnoclostridium* nov.
ERR1022368 [https://www.ebi.ac.uk/ena/data/view/ERR1022368]	Erysipelotrichaceae nov.
ERR1022338 [https://www.ebi.ac.uk/ena/data/view/ERR1022338]	*Blautia* nov.
ERR1022442 [https://www.ebi.ac.uk/ena/data/view/ERR1022442]	Ruminococcaceae nov.
ERR1022417 [https://www.ebi.ac.uk/ena/data/view/ERR1022417]	Erysipelotrichaceae nov.
ERR1022357 [https://www.ebi.ac.uk/ena/data/view/ERR1022357]	*Ruminococcus torques*
ERR1022373 [https://www.ebi.ac.uk/ena/data/view/ERR1022373]	Clostridiaceae nov.
ERR1022310 [https://www.ebi.ac.uk/ena/data/view/ERR1022310]	*Ruminococcus gnavus*
ERR1022429 [https://www.ebi.ac.uk/ena/data/view/ERR1022429]	Lachnospiraceae nov.

ERR1022326 [https://www.ebi.ac.uk/ena/data/view/ERR1022326]	*Anaerostipes hadrus*
ERR1022390 [https://www.ebi.ac.uk/ena/data/view/ERR1022390]	Lachnospiraceae nov.
ERR1022362 [https://www.ebi.ac.uk/ena/data/view/ERR1022362]	*Bacteroides faecis*
ERR1022285 [https://www.ebi.ac.uk/ena/data/view/ERR1022285]	*Sarcina* nov.
ERR1022355 [https://www.ebi.ac.uk/ena/data/view/ERR1022355]	*Oscillibacter* nov.
ERR1022346 [https://www.ebi.ac.uk/ena/data/view/ERR1022346]	*Coprococcus eutactus*
ERR1022441 [https://www.ebi.ac.uk/ena/data/view/ERR1022441]	Ruminiclostridium nov.
ERR1022366 [https://www.ebi.ac.uk/ena/data/view/ERR1022366]	*Bifidobacterium adolescentis*
ERR1022407 [https://www.ebi.ac.uk/ena/data/view/ERR1022407]	*Oscillibacter* nov.
ERR1022468 [https://www.ebi.ac.uk/ena/data/view/ERR1022468]	*Sarcina* nov.
ERR1022415 [https://www.ebi.ac.uk/ena/data/view/ERR1022415]	*Parabacteroides distasonis*
ERR1022343 [https://www.ebi.ac.uk/ena/data/view/ERR1022343]	Lachnospiraceae nov.
ERR1022402 [https://www.ebi.ac.uk/ena/data/view/ERR1022402]	Lachnospiraceae nov.
ERR1022409 [https://www.ebi.ac.uk/ena/data/view/ERR1022409]	Ruminococcaceae nov.
ERR1022427 [https://www.ebi.ac.uk/ena/data/view/ERR1022427]	*Coprococcus comes*
ERR1022273 [https://www.ebi.ac.uk/ena/data/view/ERR1022273]	*Lachnoclostridium* nov.
ERR1022296 [https://www.ebi.ac.uk/ena/data/view/ERR1022296]	*Blautia wexlerae*
ERR1022449 [https://www.ebi.ac.uk/ena/data/view/ERR1022449]	Ruminococcaceae nov.
ERR1022379 [https://www.ebi.ac.uk/ena/data/view/ERR1022379]	*Blautia luti*
ERR1022278 [https://www.ebi.ac.uk/ena/data/view/ERR1022278]	*Coprococcus* nov.
ERR1022448 [https://www.ebi.ac.uk/ena/data/view/ERR1022448]	Ruminococcaceae nov.
ERR1022283 [https://www.ebi.ac.uk/ena/data/view/ERR1022283]	*Bifidobacterium adolescentis*
ERR1022322 [https://www.ebi.ac.uk/ena/data/view/ERR1022322]	*Bacteroides uniformis*
ERR1022446 [https://www.ebi.ac.uk/ena/data/view/ERR1022446]	*Flavonifractor plautii*

ERR1022419 [https://www.ebi.ac.uk/ena/data/view/ERR1022419]	Clostridiales incertae sedis nov.
ERR1022293 [https://www.ebi.ac.uk/ena/data/view/ERR1022293]	*Roseburia inulinivorans*
ERR1022447 [https://www.ebi.ac.uk/ena/data/view/ERR1022447]	*Pseudoflavonifractor capillosus*
ERR1022386 [https://www.ebi.ac.uk/ena/data/view/ERR1022386]	Lachnospiraceae nov.
ERR1022277 [https://www.ebi.ac.uk/ena/data/view/ERR1022277]	Lachnospiraceae nov.
ERR1022331 [https://www.ebi.ac.uk/ena/data/view/ERR1022331]	*Bacteroides thetaiotaomicron*
ERR1022367 [https://www.ebi.ac.uk/ena/data/view/ERR1022367]	*Turicibacter sanguinis*
ERR1022375 [https://www.ebi.ac.uk/ena/data/view/ERR1022375]	*Romboutsia* nov.
ERR1022401 [https://www.ebi.ac.uk/ena/data/view/ERR1022401]	*Lachnospira pectinoschiza*
ERR1022482 [https://www.ebi.ac.uk/ena/data/view/ERR1022482]	*Eubacterium rectale*

* European Nucleotide Archive

Supplementary Table 4: List of strains found in female bladder but not in the vagina - from the comparison of bladder and vaginal strain collections. For further metadata on these strains refer to Supplementary Data 1.

**Species**	**ENA[*] id**	**Genome size**
Pseudomonas aeruginosa	ERR1045860 [https://www.ebi.ac.uk/ena/data/view/ERR1045860]	6035570
Gordonia terrae	ERR1203657 [https://www.ebi.ac.uk/ena/data/view/ERR1203657]	5703010
Bacillus infantis	ERR1203601 [https://www.ebi.ac.uk/ena/data/view/ERR1203601]	5204484
Klebsiella pneumoniae	ERR1045826 [https://www.ebi.ac.uk/ena/data/view/ERR1045826]	5102152
Escherichia coli	ERR1045836 [https://www.ebi.ac.uk/ena/data/view/ERR1045836]	5148822
Escherichia coli	ERR1045830 [https://www.ebi.ac.uk/ena/data/view/ERR1045830]	5013219
Escherichia coli	ERR1045825 [https://www.ebi.ac.uk/ena/data/view/ERR1045825]	5000645
Bacillus idriensis	ERR1203634 [https://www.ebi.ac.uk/ena/data/view/ERR1203634]	4598139
Enterobacter cloacae	ERR1939923 [https://www.ebi.ac.uk/ena/data/view/ERR1939923]	4655730
Enterobacter cloacae	ERR1939938 [https://www.ebi.ac.uk/ena/data/view/ERR1939938]	4654361
Enterobacter cloacae	ERR1203667 [https://www.ebi.ac.uk/ena/data/view/ERR1203667]	4649113

*European Nucleotide Archive

Supplementary Table 5: List of strains that are highly similar between female bladder and vagina whole genome datasets. For further metadata on these strains, refer to Supplementary Data 1.

Species	Bladder strain ENI* ID	Vaginal strain Assembly Accession	ANI** (%)
Actinomyces neuii	ERR1045850 [https://www.ebi.ac.uk/ena/data/view/ERR1045850]	GCA_001546135.1 [https://www.ncbi.nlm.nih.gov/assembly/GCF_001546165.1/]	98.4717
Actinomyces neuii	ERR1045813 [https://www.ebi.ac.uk/ena/data/view/ERR1045813]	GCA_001546135.1 [https://www.ncbi.nlm.nih.gov/assembly/GCF_001546165.1/]	98.6541
Lactobacillus crispatus	ERR1045852 [https://www.ebi.ac.uk/ena/data/view/ERR1045852]	GCA_000176975.2 [https://www.ncbi.nlm.nih.gov/assembly/GCF_000176975.2/]	99.2249
Lactobacillus crispatus	ERR1045847 [https://www.ebi.ac.uk/ena/data/view/ERR1045847]	GCA_000176975.2 [https://www.ncbi.nlm.nih.gov/assembly/GCF_000176975.2/]	99.7258
Lactobacillus crispatus	ERR1939956 [https://www.ebi.ac.uk/ena/data/view/ERR1939956]	GCA_000176975.2 [https://www.ncbi.nlm.nih.gov/assembly/GCF_000176975.2/]	99.1365
Lactobacillus gasseri	ERR1939928 [https://www.ebi.ac.uk/ena/data/view/ERR1939928]	GCA_000175055.1 [https://www.ncbi.nlm.nih.gov/assembly/GCF_000175055.1/]	99.3113
Lactobacillus gasseri	ERR1045824 [https://www.ebi.ac.uk/ena/data/view/ERR1045824]	GCA_000175055.1 [https://www.ncbi.nlm.nih.gov/assembly/GCF_000175055.1/]	99.4368
Lactobacillus gasseri	ERR1939936 [https://www.ebi.ac.uk/ena/data/view/ERR1939936]	GCA_000175055.1 [https://www.ncbi.nlm.nih.gov/assembly/GCF_000175055.1/]	99.4413
Lactobacillus jensenii	ERR1045855 [https://www.ebi.ac.uk/ena/data/view/ERR1045855]	GCA_000162335.1 [https://www.ncbi.nlm.nih.gov/assembly/GCF_000162335.1/]	99.8623

*European Nucleotide Archive
**Average Nucleotide Identity

Supplementary Table 6: Top 20 statistically enriched functional domains in the urogenital microbiota compared to the gastrointestinal microbiota

COG* ID	Function	Urogenital Bacteria (count)	GI Bacteria (count)	q-value
COG3425	3-hydroxy-3-methylglutaryl CoA synthase	88	0	$1.33x10^{-18}$
COG1780	Protein involved in ribonucleotide reduction	109	6	$5.76x10^{-16}$
COG0431	NAD(P)H-dependent FMN reductase	104	5	$6.52x10^{-16}$
COG4479	Uncharacterized protein YozE, UPF0346 family	72	0	$3.45x10^{-15}$
COG4473	Predicted ABC-type exoprotein transport system, permease component	71	0	$5.71x10^{-15}$
COG5503	DNA-dependent RNA polymerase auxiliary subunit epsilon	71	0	$5.71x10^{-15}$
COG3407	Mevalonate pyrophosphate decarboxylase	83	2	$8.15x10^{-15}$
COG3237	Uncharacterized conserved protein YjbJ, UPF0337 family	68	0	$2.63x10^{-14}$
COG0276	Protoheme ferro-lyase (ferrochelatase)	67	0	$4.40x10^{-14}$
COG4483	Uncharacterized protein YqgQ, DUF910 family	67	0	$4.40x10^{-14}$
COG1577	Mevalonate kinase	84	3	$5.84x10^{-14}$
COG1113	L-asparagine transporter and related permeases	112	10	$1.40x10^{-13}$
COG3759	Uncharacterized membrane protein	64	0	$2.07x10^{-13}$
COG1230	Co/Zn/Cd efflux system component	118	12	$2.53x10^{-13}$
COG2141	Flavin-dependent oxidoreductase, luciferase family (includes alkanesulfonate monooxygenase SsuD and methylene tetrahydromethanopterin reductase)	70	1	$2.74x10^{-13}$
COG4858	Uncharacterized membrane-anchored protein	60	1	$4.26x10^{-11}$
COG1257	Hydroxymethylglutaryl-CoA reductase	78	5	$5.17x10^{-11}$
COG1072	Panthothenate kinase	88	8	$9.90x10^{-11}$
COG4835	Uncharacterized protein	52	0	$1.19x10^{-10}$
COG5506	Uncharacterized protein YueI, DUF2278 family	52	0	$1.19x10^{-10}$

***** Clusters of Orthologous Groups of proteins

Supplementary Table 7: Statistically enriched functional domains in the gastrointestinal microbiota compared to the urogenital microbiota

COG[*] ID	Function	GI Bacteria (count)	Urogenital Bacteria (count)	q-value
COG1873	Sporulation protein YlmC, PRC-barrel domain family	115	3	$1.67x10^{-21}$
COG3854	Stage III sporulation protein SpoIIIAA	114	3	$2.61x10^{-21}$
COG1453	Predicted oxidoreductase of the aldo/keto reductase family	117	4	$7.85x10^{-21}$
COG2607	Predicted ATPase, AAA+ superfamily	111	3	$1.01x10^{-20}$
COG3773	Cell wall hydrolase CwlJ, involved in spore germination	112	4	$7.18x10^{-20}$
COG3546	Mn-containing catalase (includes spore coat protein CotJC)	121	7	$7.27x10^{-19}$
COG3363	Archaeal IMP cyclohydrolase	97	3	$6.47x10^{-18}$
COG1625	Fe-S oxidoreductase, related to NifB/MoaA family	94	3	$2.67x10^{-17}$
COG2155	Uncharacterized membrane protein YuzA, DUF378 family	106	6	$6.77x10^{-17}$
COG2221	Dissimilatory sulfite reductase (desulfoviridin), alpha and beta subunits	103	6	$2.54x10^{-16}$
COG4277	Predicted DNA-binding protein with the Helix-hairpin-helix motif	103	6	$2.54x10^{-16}$
COG2198	HPt (histidine-containing phosphotransfer) domain	71	0	$4.13x10^{-16}$
COG0700	Spore maturation protein SpmB (function unknown)	97	5	$5.31x10^{-16}$
COG1838	Tartrate dehydratase beta subunit/Fumarate hydratase class I, C-terminal domain	116	11	$4.30x10^{-15}$
COG1236	RNA processing exonuclease, beta-lactamase fold, Cft2 family	88	4	$4.41x10^{-15}$
COG1803	Methylglyoxal synthase	132	16	$7.87x10^{-15}$
COG1533	DNA repair photolyase	114	11	$9.59x10^{-15}$
COG1149	MinD superfamily P-loop ATPase, contains an inserted ferredoxin domain	109	10	$1.67x10^{-14}$
COG3581	Predicted nucleotide-binding protein, sugar kinase/HSP70/actin superfamily	105	9	$1.89x10^{-14}$
COG4624	Iron only hydrogenase large subunit, C-terminal domain	140	21	$1.44x10^{-13}$

***** Clusters of Orthologous Groups of proteins

Supplementary Table 8: Metadata for strains collected from the bladder and vagina of individual women. Corresponding to isolates in Figure 3.

A. Assembly statistics

Species (Subject ID)	Location	Patient ID	Relative Abundance by EQUC	Number of Contigs	N50	Median Contig Length	Average coverage
E. coli (EST08)							
ERR2092972 [https://www.ebi.ac.uk/ena/data/view/ERR2092972/]	Bladder	P1	No info	169	115344	1355	66.8
ERR2092973 [https://www.ebi.ac.uk/ena/data/view/ERR2092973/]	Vaginal	P1	No info	117	107673	9444	75.7
S. anginosus (EST14)							
ERR2092974 [https://www.ebi.ac.uk/ena/data/view/ERR2092974/]	Bladder	P2	100%	53	116687	4011	47.5
ERR2092975 [https://www.ebi.ac.uk/ena/data/view/ERR2092975/]	Vaginal	P2	100%	695	106465	265	34.9
L. iners (EST16)							
ERR2092976 [https://www.ebi.ac.uk/ena/data/view/ERR2092976/]	Bladder	P3	88%	10	99769	11783	412
ERR2092977 [https://www.ebi.ac.uk/ena/data/view/ERR2092977/]	Vaginal	P3	43%	7	839239	99769	214.0
L. crispatus (EST10)							
ERR2092980 [https://www.ebi.ac.uk/ena/data/view/ERR2092980/]	Bladder	P4	100%	238	19090	4827	395.5

ERR2092981 [https://www.ebi.ac.uk/ena/data/view/ERR2092981/]	Vaginal	P4	36%	286	20244	2877	291.8

B. **Other isolates found in each patient**

Patient	Age	HRQL*	Bladder	CFU/ml	Vaginal	CFU/ml
E. coli (EST08)	76	27.2	*Aerococcus urinae*	No info	*Aerococcus urinae*	No info
			Escherichia coli	No info	*Escherichia coli*	No info
			Gardnerella vaginalis	No info	*Klebsiella pneumoniae*	No info
			Klebsiella pneumoniae	No info	*Alloscardovia omnicolens*	No info
S. anginosus (EST14)	63	85.6	*Streptococcus anginosus*	10	*Actinobaculum schaalii*	2,000
					Actinomyces neuii	5,000
					Alloscardovia omnicolens	4,000
					Candida parapsilosis	20,500
					Corynebacterium amycolatum	400
					Corynebacterium aurimucosum	5,333
					Corynebacterium coyleae	12,000
					Enterococcus faecalis	100
					Facklamia hominis	1,500
					Globicatella sulfidiciens	200
					Gram + rods - Unknown 1	2,000
					Proteus mirabilis	250
					Streptococcus agalactiae	20,000
					Streptococcus anginosus	1,000
L. iners (EST16)	62	43.2	*Corynebacterium amycolatum*	10	*Aerococcus urinae*	100,200
			Corynebacterium aurimucosum	10	*Alloscardovia omnicolens*	3,600
			Corynebacterium imitans	10	*Corynebacterium amycolatum*	3,050
			Corynebacterium urealyticam	20	*Corynebacterium aurimucosum*	10,000
			Lactobacillus iners	400	*Corynebacterium coyleae*	1,000
					Corynebacterium simulans	1,000
					Corynebacterium tuscaniense	3,000
					Enterococcus faecalis	8,833
					Lactobacillus iners	100,000
					Staphylococcus pasteuri	400
					Staphylococcus pettenkoferi	1,000
					Unknown Gram - rods	400
L. crispatus	58	69.6	*Aerococcus urinae*	27	*Aerococcus urinae*	1,000
			Corynebacterium	10	*Arthrobacter cumminsii*	5,000

(EST10)	*coyleae*			
	Enterococcus faecalis	10	*Candida parapsilosis*	800
	Lactobacillus crispatus	45	*Corynebacterium amycolatum*	250
	Lactobacillus gasseri	20	*Corynebacterium aurimucosum*	100
	Unknown Gram + rods	10	*Corynebacterium coyleae*	300
			Enterococcus faecalis	675
			Lactobacillus crispatus	125,000
			Lactobacillus gasseri	55,350
			Micrococcus luteus	500
			Staphylococcus epidermidis	133
			Staphylococcus simulans	533
			Streptococcus anginosus	10,200
			Facklamia hominis	100,000

* Health Related Quality of Life

Supplementary Table 9: A list of the publicly available reference genomes used for comparison, downloaded from NCBI

Species	Strain	Accession
Escherichia coli	BL21	GCA_000009565.2 [https://www.ncbi.nlm.nih.gov/assembly/GCF_000009565.1/]
Escherichia coli	K12_MG1655	GCA_000005845.2 [https://www.ncbi.nlm.nih.gov/assembly/GCF_000005845.2/]
Escherichia coli	O83:H1_NRG857C	GCA_000183345.1 [https://www.ncbi.nlm.nih.gov/assembly/GCF_000183345.1/]
Escherichia coli	UPEC_CFT073	GCA_000007445.1 [https://www.ncbi.nlm.nih.gov/assembly/GCF_000007445.1/]
Lactobacillus crispatus	FB077	GCA_000301135.1 [https://www.ncbi.nlm.nih.gov/assembly/GCF_000301135.1/]
Lactobacillus crispatus	CTV-05	GCA_000165885.1 [https://www.ncbi.nlm.nih.gov/assembly/GCF_000165885.1/]
Lactobacillus crispatus	FB049-03	GCA_000301115.1 [https://www.ncbi.nlm.nih.gov/assembly/GCF_000301115.1/]
Lactobacillus crispatus	JV V01	GCA_000160515.1 [https://www.ncbi.nlm.nih.gov/assembly/GCF_000160515.1/]
Lactobacillus crispatus	125-2-CHN	GCA_000162255.1 [https://www.ncbi.nlm.nih.gov/assembly/GCF_000162255.1/]
Lactobacillus crispatus	214-1 HMP	GCA_000177575.1 [https://www.ncbi.nlm.nih.gov/assembly/GCF_000177575.1/]
Lactobacillus crispatus	ST1	GCA_000091765.1 [https://www.ncbi.nlm.nih.gov/assembly/GCF_000091765.1/]
Lactobacillus iners	DSM1335	GCA_000160875.1 [https://www.ncbi.nlm.nih.gov/assembly/GCF_000160875.1/]
Lactobacillus iners	ATCC55195	GCA_000185405.1 [https://www.ncbi.nlm.nih.gov/assembly/GCF_000185405.1/]
Lactobacillus iners	LactinV11V1-d	GCA_000149065.2 [https://www.ncbi.nlm.nih.gov/assembly/GCF_000149065.1/]
Lactobacillus iners	AB1	GCA_000177755.1 [https://www.ncbi.nlm.nih.gov/assembly/GCF_000177755.1/]
Streptococcus anginosus	C1051	GCA_000463465.1 [https://www.ncbi.nlm.nih.gov/assembly/GCF_000463465.1/]
Streptococcus	C238	GCA_000463505.1

anginosus		[https://www.ncbi.nlm.nih.gov/assembly/GCF_000463505.1/]
Streptococcus anginosus	MAS624	GCA_000478925.1 [https://www.ncbi.nlm.nih.gov/assembly/GCF_000478925.1/]
Streptococcus anginosus	SA1_831165	GCA_000831165.1 [https://www.ncbi.nlm.nih.gov/assembly/GCF_000831165.1/]
Streptococcus anginosus	SK1138	GCA_000287595.1 [https://www.ncbi.nlm.nih.gov/assembly/GCF_000287595.1/]

Supplementary Table 10: Table of Average Nucleotide Identity (ANI) values for *E. coli* isolates and reference strains

	ERR2092972	*E. coli* BL21	*E. coli* K12 MG1655	*E. coli* O83H1 NRG857C	*E. coli* UPEC CFT073
ERR2092973	99.72%	98.43%	98.56%	96.50%	96.45%
ERR2092972	---------	98.33%	98.56%	96.45%	96.43%
E. coli BL21		------------	98.16%	96.37%	96.26%
E. coli K12 MG1655			------------	96.26%	96.17%
E. coli UPEC CFT073				------------	97.31%

Supplementary Table 11: Table of Average Nucleotide Identity (ANI) values for *S. anginosus* isolates and reference strains

	ERR2092975	*S. ang* C1051	*S. ang* C238	*S. ang* MAS624	*S. ang* SA1	*S. ang* SK1138
ERR2092974	99.77%	95.32%	93.91%	93.55%	93.70%	93.06%
ERR2092975	---------	95.25%	94.0%	93.53%	93.70%	93.11%
S. ang C1051		---------	94.05%	94.09%	93.62%	93.58%
S. ang C238			---------	97.66%	93.19%	92.62%
S. ang MAS624				-----------	92.97%	92.62%
S. ang SA1					---------	94.79%

Supplementary Table 12: Table of Average Nucleotide Identity (ANI) values for *L. iners* isolates and reference strains

	ERR2092976	*L. iners* LactinV11V1d	*L. iners* DSM13335	*L. iners* AB1	*L. iners* ATCC 55195
ERR2092977	99.99%	97.29%	97.88%	97.80%	97.49%
ERR2092976	---------	97.29%	97.89%	97.81%	97.51%

L. iners LactinV11V1d	-------------	97.56%	97.46%	97.23%
L. iners DSM13335		-------------	99.95%	97.75%
L. iners AB1			---------	97.52%
L. iners ATCC 55195				----------

Supplementary Table 13: Tables of Average Nucleotide Identity (ANI) values for *L. crispatus* isolates and reference strains

	ERR 2092980	*L. crisp* ST1	*L. crisp* 2141	*L. crisp* JVV01	*L. crisp* FB049	*L. crisp* FB077	*L. crisp* CT05
ERR2092981	99.80%	96.71%	98.28%	97.99%	98.94%	99.23%	99.08%
ERR2092980	---------	96.57%	98.42%	98.18%	98.99%	99.28%	99.25%
L. crisp ST1		---------	96.06%	96.0%	96.24%	96.27%	96.35%
L. crisp 2141			---------	98.88%	98.36%	98.17%	98.11%
L. crisp JVV01				---------	98.16%	97.93%	97.82%
L. crisp FB049					---------	98.88%	98.34%
L. crisp FB077						---------	98.51%

REFERENCES

1. Group, N.H.W. *et al.* The NIH Human Microbiome Project. *Genome Res* **19**, 2317-23 (2009).
2. Browne, H.P. *et al.* Culturing of 'unculturable' human microbiota reveals novel taxa and extensive sporulation. *Nature* **533**, 543-6 (2016).
