# Supplementary material for: Culturing of female bladder bacteria reveals an interconnected urogenital microbiota
Source: Nat Commun. 2018 Apr 19;9:1557. doi: 10.1038/s41467-018-03968-5 (PMC5908796; doi:10.1038/s41467-018-03968-5)
Supplement: Supplementary file 2 — Description of Additional Supplementary Files [file 41467_2018_3968_MOESM2_ESM.pdf]

Description of Additional Supplementary Files

File Name: Supplementary Data 1
Description: List of bladder strains and metadata included in this study.
